# Cook County Medical Society—Inhalation of Medicated Vapors, &c.

**Published:** 1856-02

**Authors:** 


### EDITORIAL.

*Cook County Medical Society—Inhalation of Medicated Vapors, &c.*

At the regular meeting of the Medical Society in this city, on the evening of the 5th inst., the subject of Inhalation as a remedy for diseases of the throat and lungs, was reported on by Dr. H. A. Johnson, and discussed by the members of the society. The report stated that remedies inhaled into the air passages were capable of a two-fold action, first by contact with the mucous membrane of the air passages and cells, and second by absorption into the mass of the circulating blood. That some medicinal substances were capable of entering rapidly into the blood through the pulmonary organs, was shown by the fact that when iodine was dissolved in chloroform and inhaled with atmospheric air for five minutes, the former substance had been detected in the urine passed fifteen minutes after. The report contained extracts from several recent articles and papers published in this country and Europe, concerning different methods of inhalation, and for different purposes. A German physician had used with apparent benefit the inhalation of iodine dissolved in chloroform, for consumptive patients. The chloroform will dissolve a large proportion of iodine; and the vapor is said to partake of the soothing or sedative qualities of the former coupled with the alterant effects of the latter; a combination of qualities well calculated to produce a favorable impression on some tubercular patients. Another foreign writer recommends the inhalation of chloroform alone, as a remedy of value in the treatment of pneumonia. He relates a case treated by the chloroform inhalations exclusively, which recovered in fourteen days. Dr. A. P. Merrill, of Tennessee, recommends strongly the inhalation of iodine vapor for the relief of asthma and chronic bronchitis.

His mode of using it, is to introduce the iodine in substance into a hollow reed or piece of cane, and fill the ends with fine wool; and then let the patient breathe through this. The warmth imparted by the hand which holds the cane and by the expired air induces a sufficiently rapid evaporation of the iodine, while the wool tends to equalize the temperature of the inhaled air before it reaches the lungs. A writer in the St. Louis *Medical and Surgical Journal*, recommends the use of opiate and narcotic inhalations for the relief of neuralgia, as more efficacious than remedies taken into the stomach. In concluding his report, Dr. Johnson, said, he had used inhalations of iodine sometimes alone, and sometimes with camphorated tincture of opium, in cases of chronic bronchial diseases, and with decided benefit. But he coincided with the opinion expressed by Dr. Henry Goadby, in a recent article published in the *Peninsular Journal*, that in true tubercular diseases of the lungs, all inhalations were nearly if not quite useless.

In the course of the discussion which followed the report, Dr. Bevan remarked that he had in his practice resorted to the inhalation of medicated vapors to a very limited extent only. He had a patient under treatment with tubercular disease of the lungs so far advanced as to present cavities of considerable size.

During the last four or five days he had directed the cautious inhalation of iodine vapor mixed with benzoic acid; and thus far it had lessened the amount of expectoration and palliated the symptoms. Dr. Varian thought that, in those cases of tubercular phthisis coupled with more or less pneumonic inflammation, the inhalation of chloroform might be beneficial, but not in such advanced cases as are accompanied by cavities and anemia. Dr. Kluber, thought the inhalation of chloroform more dangerous than the pneumonic inflammation for which it had been recommended. The general sentiment of the society, so far as could be gathered from the discussion, was that the inhalation of medicated vapors might be very servicable in many cases of chronic diseases of the fauces, larynx, and bronchial mucous membrane, but is at best only palliative in tubercular diseases of the lungs. It is to be hoped that the very general attention which has recently been given to this ancient method of treating affections of the respiratory organs, will result in some more definite and reliable rules in regard to the kind of substances to be used, and the particular classes of cases which they are calculated to benefit. After the discussion the society listened to a very interesting paper on hysteria, by Dr. Heydock, which we cannot analize at present.

